# Utilizing surface plasmon resonance as a novel method for monitoring *in-vitro* P-glycoprotein efflux

**DOI:** 10.3389/frbis.2024.1367511

**Published:** 2024-03-08

**Authors:** Phuong H. Nguyen, Shuolin Cui, Amanda M. Kozarich, Alex Rautio, Arthur G. Roberts, May P. Xiong

**Affiliations:** Department of Pharmaceutical and Biomedical Sciences, University of Georgia, Athens, GA, United States

**Keywords:** p-glycoprotein, surface plasmon resonace, efflux kinetics, efflux analysis, transport assay

## Abstract

P-glycoprotein (Pgp) is known for its dichotomous roles as both a safeguarding efflux transporter against xenobiotics and as a catalyst for multidrug resistance. Given the susceptibility of numerous therapeutic compounds to Pgp-mediated resistance, compliance with Food and Drug Administration (FDA) guidelines mandates an in-depth *in vitro* transport assay during drug development. This study introduces an innovative transport assay that aligns with these regulatory imperatives but also addresses limitations in the currently established techniques. Using Pgp-reconstituted liposomes and employing surface plasmon resonance (SPR), this study developed a distinct method of measuring the relative transport rates of Pgp substrates in a controlled microenvironment. The Pgp substrates selected for this study—quinidine, methadone, and desipramine—resulted in transport ratios that corroborate with trends previously observed. To assess the kinetics of Pgp-mediated transport, the results were analyzed by fitting the data to both currently proposed Pgp substrate translocation models—the vacuum cleaner and flippase models. While the resulting kinetic analysis in this study lends support predominantly to the vacuum cleaner model, this study most notably developed a novel method of assessing Pgp-mediated transport rates and real-time kinetics using surface plasmon resonance.

## 1 Introduction

P-glycoprotein (Pgp), a transmembrane transporter, functions as a protective mechanism within the human biological system by expelling xenobiotics from tissues. However, Pgp’s involvement in multidrug resistance in cancer and other diseases is well-documented. Most small molecule therapeutics are Pgp substrates, leading to their efflux by Pgp at therapeutic doses and thereby complicating treatment. Efforts to counter this challenge involve designing non-Pgp substrate small molecules. A fundamental step in this endeavor is measuring the transport rate of Pgp for specific compounds and determining their status as Pgp substrates. Given the critical role of assessing Pgp substrate characteristics in novel compound development, the Food and Drug Administration (FDA) issued a guideline in January 2020 named “*In vitro* drug interaction studies-cytochrome P450 enzyme-and transporter-mediated drug interactions guidance for industry”. This guideline outlines acceptable *in vitro* systems and methodologies, including the use of Caco-2 cells, commercial or in-house vesicles, knock-out/down cells, and transfected cells, for investigating Pgp transport rates (Brum, 2020).

Presently, three widely used methods adhere to the outlined guidelines for quantifying Pgp efflux. The initial approach involves a vesicle transport assay utilizing inside-out vesicles (Ambudkar et al., 1998; Sharom et al., 1999; Glavinas et al., 2008; Gameiro et al., 2017; Toyoda et al., 2019). This method gauges the substance’s permeation into vesicles when exposed to Pgp and a Pgp inhibitor. A quantifiable substrate, often radioactively or fluorescently labeled, is employed (Ambudkar et al., 1998; Sharom et al., 1999; Glavinas et al., 2008). Advanced quantification may also involve mass spectrometry (Takada et al., 2018), although both quantification techniques follow the assay endpoint and might not precisely capture real-time Pgp transport. The second method entails Pgp overexpression in systems like Caco-2 or MDCK cells (Silva et al., 2015a; Silva et al., 2015b; Gameiro et al., 2017). Dubbed the drug resistance/accumulation test, it observes how chemicals penetrate and impact cell viability in the presence of Pgp or its inhibitor. A discernible split in the cell viability curve between Pgp inhibitor absence and presence designates a Pgp substrate (Silva et al., 2015a; Silva et al., 2015b). However, this method’s drawback lies in its use of a mammalian cell system containing diverse transporters, yielding cumulative effects rather than strict Pgp specificity. The third method for Pgp efflux measurement employs a transcellular system utilizing Pgp overexpressing cells (Matsumoto et al., 2018; Ohashi et al., 2019). A cell monolayer is established on one side of the transwell system, assessing compound transport across to the other side. Like the previous method, this approach may encounter interference and cross-reactivity from other cellular transporters.

In addition to efflux rate, understanding the transport kinetics of a specific compound through Pgp is vital for comprehending its transport mechanism, ultimately aiding in improved drug design to circumvent Pgp. However, a more comprehensive grasp of Pgp’s transport process kinetics is required. Existing studies on Pgp kinetics predominantly relate to substrate-coupled ATP hydrolysis and employ Michaelis-Menten analysis (Borgnia et al., 1996; Boulton et al., 2002). While valuable for comparing how diverse substrates influence ATP hydrolysis in Pgp, this analysis typically yields substrate Km and Vmax values but falls short of revealing the complete transport mechanism. Furthermore, certain biochemical and biophysical investigations delve into the kinetics of substrate partitioning into the membrane preceding Pgp interaction (Romsicki and Sharom, 1999; Clay and Sharom, 2013). A comprehensive examination of the full transport process kinetics, encompassing substrate-membrane and substrate-Pgp interactions, still merits deeper investigation.

This study aimed to employ surface plasmon resonance (SPR) to gauge the efflux rate of Pgp and elucidate the transport kinetics of Pgp with distinct substrates. The SPR-based transport assay aligns with FDA guidelines for *in vitro* transporter investigations and addresses limitations of current methods. Additionally, the transport kinetics analysis via SPR offers insights into both substrate-membrane and substrate-Pgp interactions, thereby enhancing our understanding of Pgp’s transport mechanism.

## 2 Materials and methods

### 2.1 Materials

Quinidine was sourced from ThermoFisher Scientific (Waltham, MA), while methadone hydrochloride came from the U. S. Pharmacopeia (Rockville, MD). Rhodamine B, desipramine hydrochloride, and ADP sodium were procured from Sigma-Aldrich (St. Louis, MO). Cholesterol, tris-HCl, and disodium ATP were obtained from Amresco (Solon, OH). Dithiothreitol (DTT) was purchased from Gold Biotechnology (Olivette, MO), and ethylene glycol tetraacetic acid (EGTA) and imidazole were sourced from Alfa Aesar (Tewksbury, MA). *Escherichia* (*E*.) *coli* total extract lipid and *E. coli* polar extract lipid were acquired from Avanti Polar Lipids Inc. (Alabaster, AL). The detergent n-dodecyl-β-D-maltoside (DDM) was procured from MilliporeSigma (Formerly, EMD Millipore Corporation) (Burlington, MA), and 4-(2-hydroxyethyl)−1−piperazineethanesulfonic acid (HEPES) and acrylamide from MilliporeSigma (Formerly, Calbiochem) (Burlington, MA). Sodium orthovanadate (Na_3_VO_4_) was sourced from Enzo Life Sciences (Farmingdale, NY). All other chemicals were acquired from ThermoFisher Scientific (Waltham, MA).

### 2.2 Expression, purification, and reconstitution of Pgp

The his-tagged wild-type mouse Pgp (Abcb1a, MDR3) was purified from *Pichia* (*P*.) *pastoris* using affinity chromatography with nickel-nitrilotriacetic acid (Ni-NTA) (Thermo Fisher Scientific) followed by diethylaminoethyl cellulose (DEAE) resin (Thermo Fisher Scientific) as previously described (Lerner-Marmarosh et al., 1999; Bai et al., 2011). Briefly, Pgp solubilized by DDM was reconstituted into 200 nm liposomes using a previously established procedure so that the orientation of the transporter was inside-out with nucleotide-binding domains projected outside of the liposome (Ledwitch et al., 2016; Nguyen et al., 2020). Liposomes were prepared using 80% wt/vol *E. coli* Polar Lipid Extract (Avanti Polar Lipids) and 20% wt/vol cholesterol. A thin lipid film was created by evaporating the mixture of lipid extract and cholesterol dissolved in chloroform using a rotorvap system for 1 h. Rehydration of the dried lipid film in an aqueous phase (0.1 mM EGTA and 50 mM Tris-HCl) was followed by ten cycles of freeze-thaw in liquid nitrogen, generating liposomes of various sizes. The unilamellar liposomes with an average size of 200 nm were created by extruding the multilamellar liposome eleven times through a 400 nm filter with a LIPEX extruder. Bigger liposome size might lower SPR signal and decrease the signal sensitivity because analytes used are relatively small compared to ligand. Detergent-solubilized Pgp was dialyzed in HEPES buffer (20 mM HEPES, 100 mM NaCl, 5 mM MgCl_2_, 2 mM DTT, pH 7.4) for 2 h to remove DDM and glycerol. Dialyzed-protein was incubated with the extruded liposome with a lipid-to-protein ratio of 6.25 μM Pgp (mg ml^−1^ lipid)^−1^ for an hour and dialyzed for another 2 h to make proteoliposomes. The concentration of protein reconstituted in the liposomes was determined using a DC Protein Assay Kit II (Bio-Rad, Hercules, CA).

### 2.3 Vesicle transport assay

The ability of Pgp-embedded liposomes to transport pharmaceutical agents is conducted using a dialysis tube in Chifflet buffer (150 mM NH_4_Cl, 5 mM MgSO_4_, 0.02% wt/vol NaN_3_, 50 mM Tris-HCl, pH 7.4) (Cui et al., 2022). Briefly, 1 mg/mL of liposome (control) or proteoliposome suspension in Chifflet buffer was transferred into narrow dialysis tubing (3.5 kDa MWCO), which was then placed into 50 mL centrifuge tubes containing 20 mL of Chifflet buffer added with 2 mg of rhodamine B and with or without 5 mM of ATP. Then, the tubes were incubated at 37 °C for up to 2 h. At time points 0, 30 min, 60 min, and 120 min, 200 μL of the buffer outside the dialysis tubing was collected, and 200 μL of fresh buffer was added to the tubes to maintain a total of 20 mL release media. The collected samples had their absorbances tested at 550 nm using UV-vis spectroscopy. The study was carried out in triplicate, and samples were evaluated through a standard curve created successive 2-fold dilutions of free rhodamine B (R^2^ = 0.9987) to calculate the ratio of absorbance to rhodamine B concentration.

After calculating rhodamine B concentrations using absorbance through the standard curve, the transport efficiency was calculated using the following equation. Briefly, concentration (I) is the initial concentration of rhodamine B in the buffer, while concentration (S) is the concentration of rhodamine B in the buffer at each time point. Reduced values indicate the drug being transported into the proteoliposome.


(1) $$\text{Transport}\;\text{efficiency}\;(\%)=\frac{\text{RhoB}\;\text{concentration}\;(I-S)}{\text{RhoB}\;\text{concentration}\;(I)}\times 100$$

### 2.4 ATPase assay

ATPase assays were carried out to measure the ATP hydrolysis of Pgp in the presence of three different substrates, including desipramine, methadone, and quinidine. The Chifflet method, which detects the formation of inorganic phosphate following ATP hydrolysis, was used as described previously (Ledwitch et al., 2016; Gibbs et al., 2018; Nguyen et al., 2020). The ATPase activity of the three drugs was measured in the presence of 50 nM Pgp reconstituted in liposomes in Chifflet buffer.

The ATPase activity of desipramine, methadone, and quinidine were fit by nonlinear regression using Igor Pro 6.2 software (Wavemetrics, Tigard, OK) as described previously (Ledwitch et al., 2016; Wilt et al., 2017; Nguyen et al., 2020). The ATP hydrolysis kinetics of desipramine and methadone fit with the monophasic equation derived from the Michaelis-Menten equation, where *v* is the ATP hydrolysis rate, *V*_max_ is the maximum ATP hydrolysis rate at saturating drug, [*L*] is the ligand concentration, *K*_*m*_ is the Michaelis-Menten constant, and *v*_*basal*_ is the basal ATPase activity in the absence of the drug.


(2) $$v=\frac{v_{\text{max}}\;[L]}{K_{m}+[L]}+v_{\text{basal}}$$

Unlike the ATP hydrolysis kinetics of methadone and desipramine, quinidine established a biphasic ATPase activity profile. Substrate inhibition was used for biphasic kinetics, where *K_I_* is the inhibitory constant.


(3) $$v=\frac{v_{\text{max}}}{1+\frac{K_{m}}{\left[L\right]}+\frac{\left[L\right]}{K_{I}}}+v_{\text{basal}}$$

### 2.5 Surface plasmon resonance

Surface plasmon resonance is typically used to measure the real-time kinetics of the interaction between two molecules. The classic SPR monitors kinetic events by measuring the change in refractive angle of the incident light upon changes on the sensor surface (Myszka, 1997). The sensor in the classic SPR system is normally made of gold, which exhibits a broad plasmonic field. The SPR system we used in this study is a localized SPR (LSPR) from Nicoya Lifescience (Canada) instead of a classic SPR. Localized SPR monitors the change in absorbance wavelength at the local point where binding events occur instead of the change in the refractive angle (Hutter and Fendler, 2004; Willets and Van Duyne, 2007). The sensor of the LSPR system is made of nanogold, which has a smaller plasmonic field than a gold film and is therefore sensitive to localized changes instead of bulk changes. LSPR is known to have similar sensitivity for biomolecular binding events to SPR but is less susceptible to drift due to bulk changes (Willets and Van Duyne, 2007).

**FIGURE 1:**
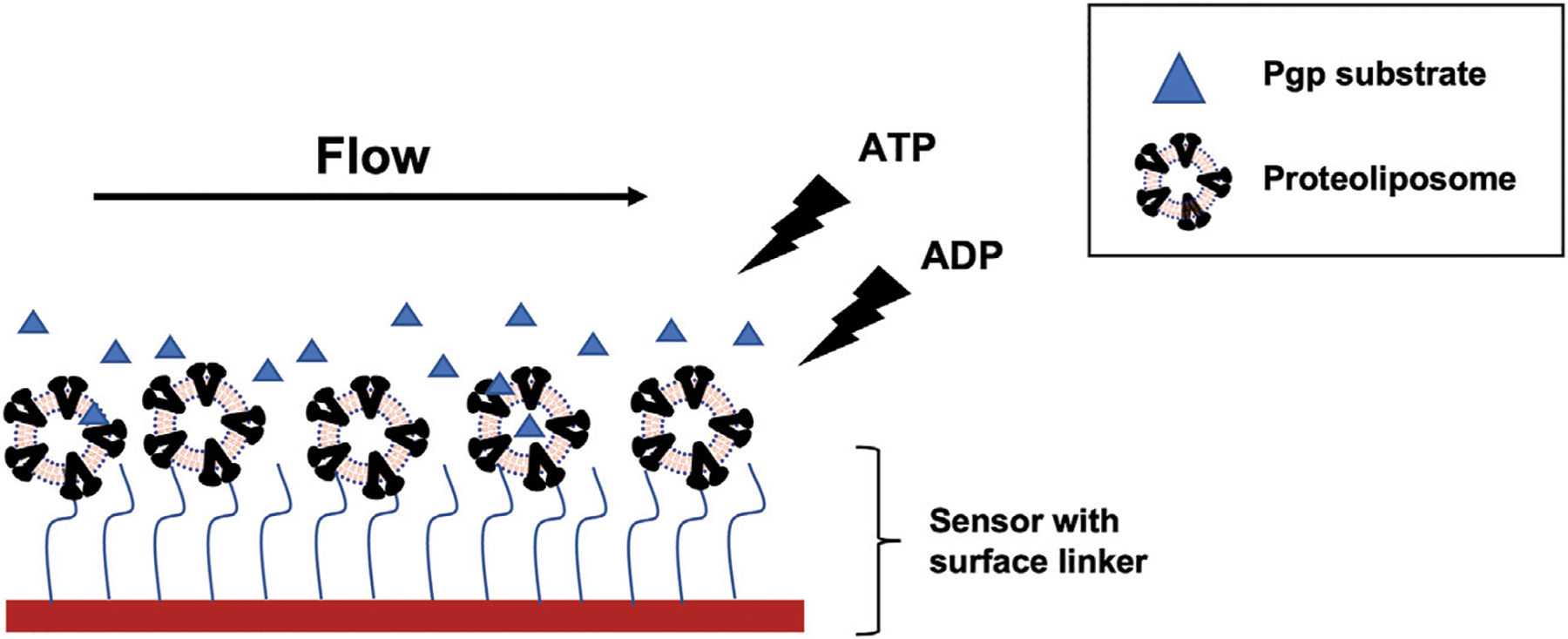
SPR experiment scheme with Pgp reconstituted into liposome and Pgp substrates (blue triangle). Proteoliposome was captured onto liposome sensor or NTA sensor. The liposome sensor has a hydrophobic chain (blue wavy line) that can insert into the bilayer. NTA sensor has NTA residue (blue wavy line) that can bind to His tag on Pgp. The substrate flowed on the sensor surface after immobilization with ATP, ADP, or no nucleotide. Detection area (red block) on the flow cell for each channel measures a spot of 1 mm × 1 mm.

The SPR experiment was carried out with the three drugs, desipramine, methadone, and quinidine, in the presence of 5 mM ATP, 5 mM ADP, or no nucleotide as analytes shown in Figure 1. Analyte concentrations used were 500, 250, 125, 62.5, and 31.25 *μM*. Pgp reconstituted in liposomes was used as the ligand and were immobilized onto the liposome or NTA sensor at a saturated level, which typically takes two injections of the ligand at 20 μM of the protein. The Liposome sensor has a hydrophobic chain to capture the bilayer portion of the proteoliposome, while the NTA sensor captures the His tag on Pgp. The running buffer contained 100 mM Tris, 150 mM NaCl, 5 mM MgCl_2_, 0.5% DMSO, and pH 7.4. The running buffer contained the same amount of nucleotide (ATP, ADP, or no nucleotide) as in the analyte solution. The association phase was set to 1,200 s and the dissociation phase was set to 800 s. All the experiments were performed at a speed of 5 *μl/min*. Regeneration of the liposome sensor was carried out using 20 mM CHAPs, while the regeneration of the NTA sensor was carried out using 300 mM EDTA and 250 mM imidazole. Double referencing was carried out and subtracted from the data before further analysis.

### 2.6 Data fitting using COPASI

COPASI is the software used previously by other studies to fit complex kinetics data. SPR data with multiple reactions and compartments were also successfully modeled using COPASI (Collier, 2012; Kim et al., 2012). Therefore, we used this software to fit the transport data obtained from the SPR experiment. The data were fit with two models, the vacuum cleaner and the flippase model. Each model consists of five different compartments, including bulk solvent, the sensor’s surface, the liposome’s outer leaflet, the liposome’s inner leaflet, and the liposome’s inner space. There are six reactions used in each model to represent the whole transport process. Specifically, in the vacuum cleaner model, the following reaction was used: (Brum, 2020): analyte diffused from the bulk to the surface of the sensor, (Ambudkar et al., 1998), analyte diffused from outside to outer leaflet of the proteoliposome, (Gameiro et al., 2017), analyte bound to Pgp, (Glavinas et al., 2008), Pgp transported analyte into the inner space of the proteoliposome, (Sharom et al., 1999), analyte diffused from the outer leaflet to the inner leaflet of the proteoliposome, (Toyoda et al., 2019), analyte diffused from the inner leaflet to the inner space of the proteoliposome. The flippase model used similar reactions except for reaction (Glavinas et al., 2008), in which Pgp transported the analyte to the inner leaflet instead of the inner space of the proteoliposome.

The global quantities were defined as the SPR output signal, represented by the following equation:

(4) $$RU=k\left({\left[SIN\right]}_{t}+{\left[SLO\right]}_{t}+{\left[\text{SPGP}\right]}_{t}+{\left[SLI\right]}_{t}\right)+C$$

Where *RU* is the relative unit in SPR sensorgram, *k* is the overall scaling, *C* is the baseline constant, [*SIN*]_*t*_, [*SLO*]_*t*_, [*SPGP*]_*t*_, and [*SLI*]_*t*_ represent the time-dependent evolution of the analytes incorporated into the inner space of the proteoliposome, the outer leaflet of the proteoliposome, the Pgp, and the inner leaflet of the proteoliposome, accordingly. A time course kinetics simulation of the SPR signal was carried out with intervals of 50,000 and an interval size of 0.06 s for a duration of 3,000 s using the deterministic method. Following the simulation, parameter estimation was used to fit the experimental SPR data. All available convergence methods were implemented, but the best result was obtained with an evolutionary programming algorithm. Data with different concentrations of the analytes were fit globally with the kinetics for each part of Equation 4, varied freely. The scaling factor *k* and baseline constant C were fitted locally for each concentration. As indicated in the previous AFM study, the amount of Pgp reconstituted into the liposome has 80% inside-out orientation (Sigdel et al., 2018). Only Pgp with the nucleotide binding domains projecting out of the proteoliposome has access to ATP and can efflux substrates. Therefore, a restraint was applied to the number of Pgp involved in active transport during simulation.

## 3 Results

### 3.1 Vesicle transport assay as a standard transport method showed that the Pgp substrate was transported inside the proteoliposome by Pgp

**FIGURE 2:**
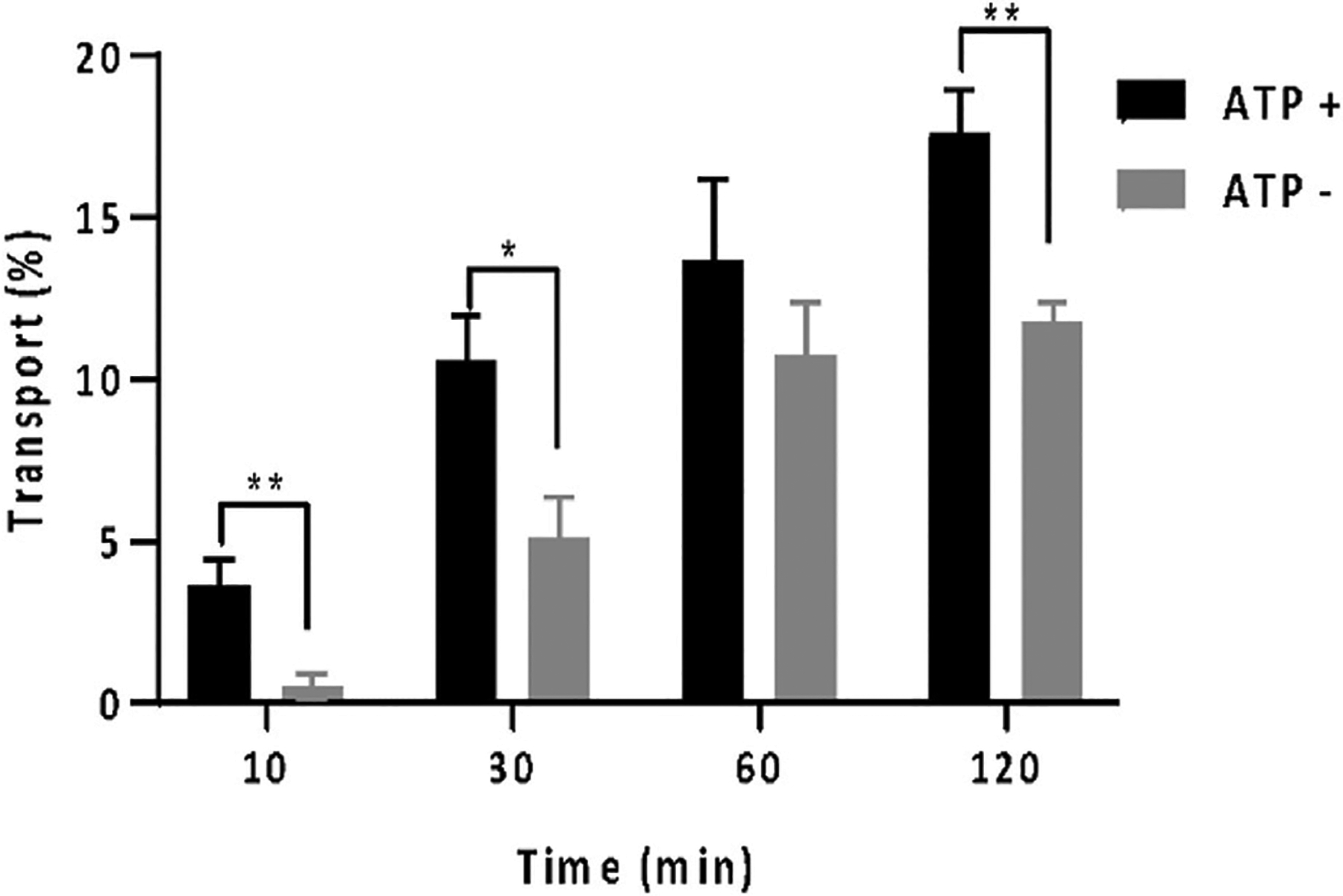
*In vitro* vesicle transport assay with rhodamine B with the percentage of rhodamine which was located in the proteoliposome at different time points: 10, 30, 60, and 120 min. The amount of rhodamine B was monitored by absorbance at 550 nm. The gray bar shows the data when there was no ATP present. The black bar shows the data when 5 mM of ATP was present. One asterisk indicates *p* ≤ 0.05. Two asterisks indicate *p* ≤ 0.01. n=3. Error bar is ±SD.

Utilizing rhodamine B, a well-established Pgp substrate (Sarver et al., 2002; Loetchutinat et al., 2003), the vesicle transport assay was conducted. The transport efficiency of rhodamine B was depicted in Figure 2, illustrating rates at 10, 30, 60, and 120 min into the assay. Rhodamine B is recognized for its Pgp-mediated membrane transport facilitated by ATP (Loetchutinat et al., 2003). Our findings demonstrated a substantial increase in rhodamine B accumulation within proteoliposome in the presence of ATP compared to its absence. Notably, within 10 min, rhodamine B uptake without ATP was around 5%, whereas with 5 mM ATP, it escalated to 10%, with statistical significance. Over 120 min, ATP-assisted transport raised vesicular rhodamine B content to 17%, while this remained at 12% without ATP. Rhodamine uptakes in proteoliposome in the absence of ATP is due to passive diffusion. These outcomes highlight successful Pgp-mediated transport of rhodamine B within proteoliposome with ATP support, validating the functionality of the employed inside-out vesicle system. ATPase of methadone, quinidine, and desipramine confirms the activity of the transporter.

**FIGURE 3:**
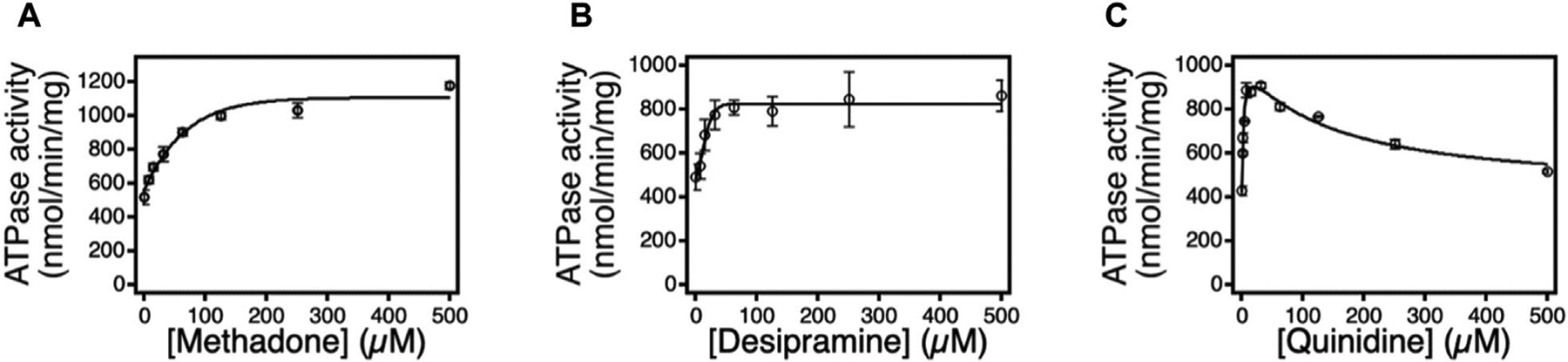
The effects of methadone, desipramine, and quinidine on Pgp mediated ATP hydrolysis. ATPase activity in response to varying concentrations of **(A)** methadone, **(B)** desipramine, and **(C)** quinidine. The points represent the average of at least three experiments, and the error bars reflect the standard deviation. Fits to the points in Eq. 1 and (Ambudkar et al., 1998) are shown as solid lines. n=3. Error bar is ±SD.

To further assess proteoliposome activity, an ATPase assay was conducted using three additional Pgp substrates: methadone, quinidine, and desipramine. Figure 3 depicts the ATP hydrolysis kinetics curve for each substrate across varying concentrations. The ATPase data reveals that, in the presence of these drugs, Pgp’s basal ATP hydrolysis rate measured 482 ± 50 nmolmin^−1^mg^−1^, consistent with prior investigations (Gibbs et al., 2018; Nguyen et al., 2020). The presence of sodium orthovanadate, a non-competitive ATP inhibitor, resulted in a decline of Pgp’s ATPase activity to approximately 100 nmolmin^−1^mg^−1^, corroborating findings from previous studies (Ledwitch et al., 2016; Wilt et al., 2017).

Methadone incited monophasic ATP hydrolysis activation in Pgp, revealing kinetics with a peak of approximately 800 nmolmin^−1^mg^−1^ at saturating methadone levels. Curve fitting yielded *v*_*max*_ and *K*_*m*_ values of 849 ± 45 nmolmin^−1^ mg^−1^ and 28 ± 10 μM, respectively, consistent with a prior methadone-Pgp study (Gibbs et al., 2018). Similarly, desipramine mirrored methadone’s behavior, eliciting monophasic ATPase activity and implying a solitary binding site on the transporter for both compounds. In the presence of desipramine, Pgp’s ATPase kinetics revealed *v*_*max*_ and *K*_*m*_ values of 904 ± 65 nmolmin^−1^mg^−1^ and 12 ± 4 μM, respectively. Notably, this study uniquely unveils the ATP hydrolysis kinetics of Pgp influenced by desipramine, as no prior research reported *v*_*max*_ and *K*_*m*_ values for this interaction.

In contrast to methadone and desipramine, quinidine induced Pgp’s ATP hydrolysis in a biphasic manner, implying the presence of at least two quinidine binding sites on Pgp, as observed with other substrates like verapamil, lidocaine, and loperamide. The kinetics demonstrated a peak at approximately 900 nmolmin^−1^mg^−1^ at a quinidine concentration of 15.6 μM. Fitting the biphasic kinetics curve to Equation 3 yielded *v*_*max*_, *K*_*m*_, and *K*_*I*_ values of 626 ± 44 nmolmin^−1^mg^−1^, 2.9 ± 1 μM, and 131 ± 34 μM, respectively. Notably, the *K*_*m*_ and *v*_*max*_ values obtained in this study were consistent with those reported in a prior investigation involving quinidine and Pgp (Matsunaga et al., 2006).

### 3.2 SPR signal amplitude correlated accordingly to the transport rate of each Pgp substrate

The SPR signal amplitude corresponds to alterations in mass on the sensor surface (Myszka, 1997). Consequently, the signal amplitude can serve to monitor changes in mass within the proteoliposome. Should the analyte occupy the proteoliposome, this would elevate weight, translating into an increased signal amplitude. Leveraging this SPR attribute, we can gauge the relative transport rate of the Pgp substrate by observing fluctuations in signal amplitude. Active Pgp-mediated transport, facilitated by ATP, is expected to yield greater substrate accumulation within the proteoliposome than in the absence of ATP. This heightened substrate accumulation within the proteoliposome would generate a more pronounced SPR signal.

**FIGURE 4:**
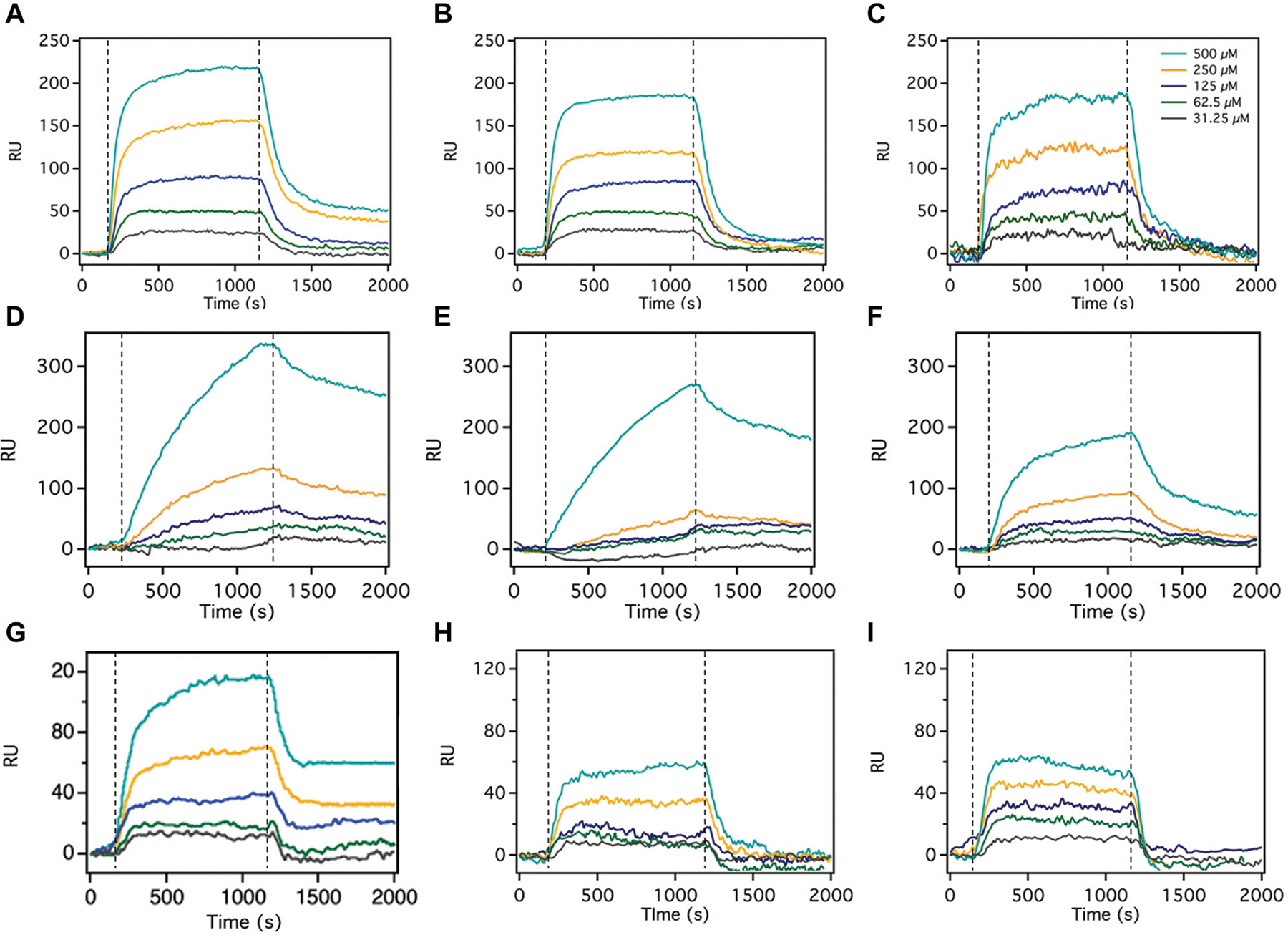
The SPR signals of desipramine, methadone, and quinidine in the presence of 5 mM ATP, no nucleotide, and 5 mM ADP. The association phase is 1200s, and the dissociation phase is 800s, both injected at a 5 μL/min speed. The SPR sensorgrams for each Pgp substrate comprised 5 different substrate concentrations: 500, 250, 125, 62.5, and 31.25 μM. **(A)** is the SPR signal of desipramine with 5 mM ATP, **(B)** is desipramine without nucleotide, and **(C)** is desipramine with 5 mM ADP. **(D)** is the SPR signal of methadone with 5 mM ATP, **(E)** is methadone without nucleotide, and **(F)** is methadone with 5 mM ADP. **(G)** is the SPR signal of quinidine with 5 mM ATP, **(H)** is quinidine without nucleotide, and **(I)** is quinidine with 5 mM ADP. Dashed line represents start of association phase and dissociation phase. Figure shows the representative curve of each experiment, the number of replicates for each experiment is n=5.

Experiments involving three distinct Pgp substrates consistently demonstrated that the SPR signal amplitude in the presence of 5 mM ATP significantly surpassed the amplitude in the absence of nucleotides or with ADP (Figure 4). The ratio between SPR amplitude in the presence of ATP and that without nucleotides is an indicator of each substrate’s relative transport rate. Table 1 presents the ratios for quinidine, methadone, and desipramine as 1.64 ± 0.12, 1.31 ± 0.1, and 1.30 ± 0.08, respectively. These ratios align with prior investigations that assessed Pgp transport rates of these three compounds using a cell-based system. Mahar et al. and Tournier et al. reported Pgp efflux rates of 2 for desipramine and methadone (Doan et al., 2002; Tournier et al., 2011). However, quinidine’s reported Pgp efflux rate was 10 (Patil et al., 2011). This concurs with the SPR amplitude findings, indicating that quinidine exhibits significantly higher transport rate than methadone and desipramine, while the latter two share the same transport rate. The larger difference in sensitivity between *in vitro* cell-based assay and SPR assay is because the design of the SPR assay involved large molecular weight ligand and small molecular weight analyte, which decreased the overall signal sensitivity. However, SPR assay could detect the transport of weak substrates like methadone and desipramine suggesting that the assay is sufficient for screening Pgp efflux. Hence, the SPR signal amplitude proves valuable in monitoring the relative Pgp transport rate across diverse compounds.

### 3.3 The kinetics of SPR transport of desipramine supported the vacuum cleaner model for the Pgp transport mechanism

**FIGURE 5:**
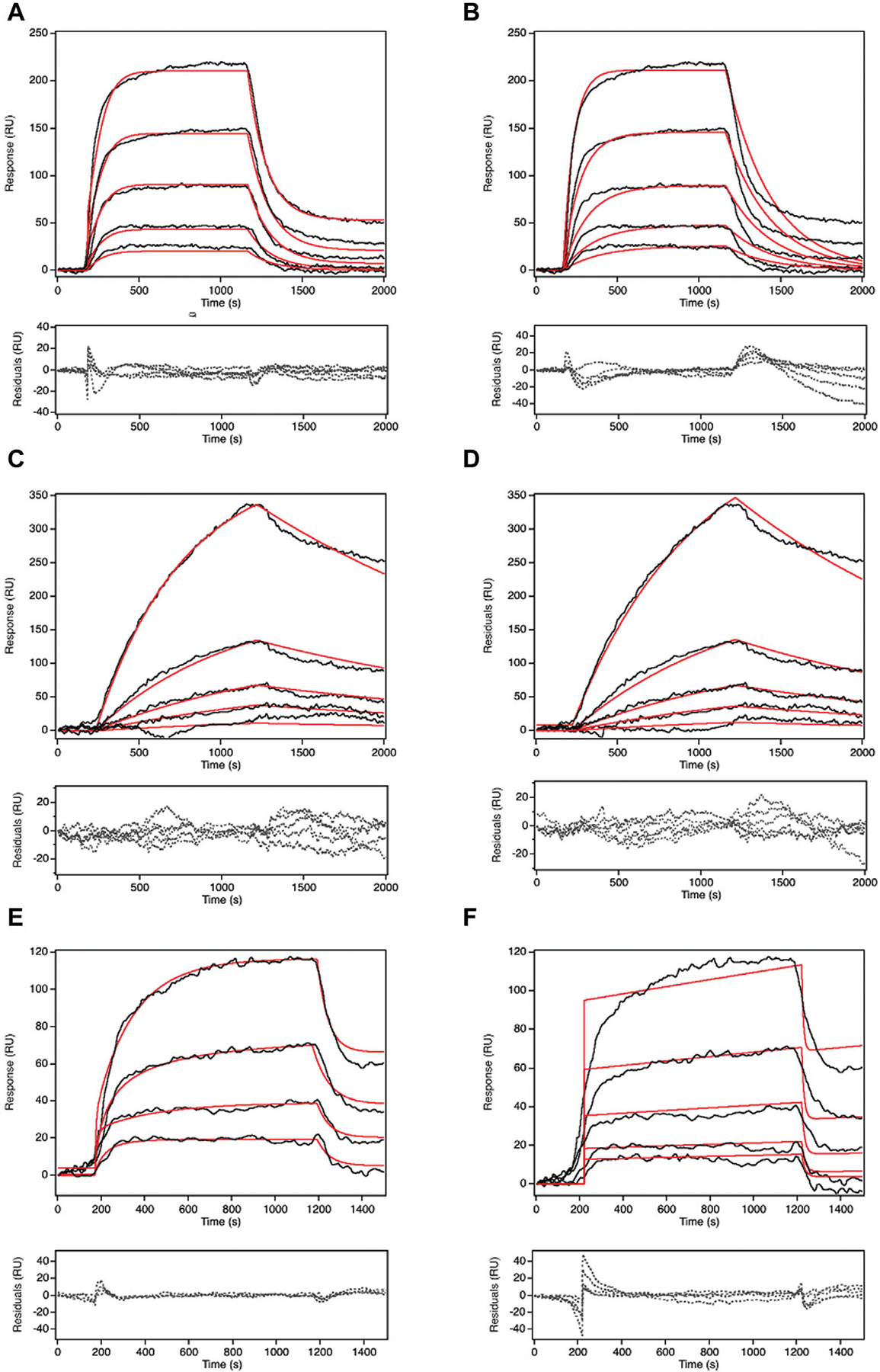
COPASI fitting of SPR signal in the presence of 5 mM ATP at 500 μM of the substrates with two different models: vacuum cleaner and flippase. The black line is the experimental SPR data. The red line is the fitted line. The dashed line is the residual of the fit. **(A)** is the fitting for desipramine with vacuum cleaner model, **(B)** is the fitting for desipramine with flippase model, **(C)** is the fitting for methadone with vacuum cleaner model, **(D)** is the fitting for methadone with flippase model, **(E)** is the fitting for quinidine with vacuum cleaner model, **(F)** is the fitting for quinidine with flippase model. Figure shows the representative curve of each experiment, the number of replicates for each experiment is n=5.

Recognized for its capability to track real-time binding kinetics, SPR serves as a means to not only gauge Pgp’s relative transport rate across diverse compounds but also to delve into the transport kinetics of each compound (O’Shannessy, 1994). Presently, two prominent models for Pgp transport, the vacuum cleaner and flippase models have gained acceptance (Romsicki and Sharom, 2001; Sharom, 2014; Kim and Chen, 2018). The SPR data were subjected to fitting using both models. Figure 5 portrays the fittings and residuals for the desipramine dataset, revealing a distinct preference for the vacuum cleaner model over the flippase model. This outcome signifies that the SPR-derived desipramine transport data align more favorably with the vacuum cleaner model, thus suggesting its support for the vacuum cleaner transport mechanism.

The kinetics of each reaction encompassed in the transport process of the three drugs were also assessed, as outlined in Table 2. The values of k_on_ and k_off_ for the reaction involving substrate diffusion from the exterior to the outer leaflet exhibited remarkable similarity for desipramine (within the 10^−6^ M^−1^s^−1^ and 10^−6^ s^−1^ range), suggesting a relatively balanced rate for this bidirectional process in desipramine. Conversely, in the case of methadone, the on-rate (5.07 × 10^−5^ M^−1^s^−1^) for diffusion from the exterior into the outer leaflet slightly exceeded the off-rate (8.34 × 10^−6^ s^−1^), implying a slight bias against methadone’s diffusion into the outer leaflet. For quinidine, the k_on_ for diffusion into the outer leaflet of the proteoliposome was 4.62 × 10^−8^ M^−1^s^−1^, while the k_off_ was 1.20 × 10^−5^ s^−1^, indicating a greater likelihood for quinidine to stay in the exterior than to diffuse into the outer leaflet of the bilayer.

In the reactions involving desipramine’s diffusion from the outer leaflet to the inner leaflet and the departure of substrate from the outer leaflet to bind to Pgp, the k_on_ was significantly lower than the k_off_. This observation suggests a preference for the desipramine-outer leaflet complex over the desipramine-inner leaflet and desipramine-Pgp complex. Similar trends were observed with both methadone and quinidine, indicating that all three drugs displayed a preference for complex formation with the outer leaflet rather than with Pgp or the inner leaflet of the bilayer.

Remarkably, the reactions in which Pgp facilitated the transport of substrates inside the vesicle exhibited significantly smaller k_off_ values (6.44 × 10^−8^ s^−1^ for desipramine, 1.89 × 10^−4^ s^−1^ for methadone, and 7.91 × 10^−8^ for quinidine) compared to the corresponding k_on_ values (3.57 × 10^−5^ M^−1^s^−1^ for desipramine, 2.75 × 10^−4^ M^−1^s^−1^ for methadone, and 1.6 × 10^−3^ M^−1^s^−1^ for quinidine), indicating a pronounced preference for substrate transportation into the proteoliposome interior. In the final reaction, involving substrates diffusing from the inner leaflet to the inner space of the proteoliposome, the *k*_*on*_ rates were significantly lower than the *k*_*off*_ rates for all three drugs. This observation suggests that all three substrates preferred the hydrophobic inner leaflet over the hydrophilic core of the proteoliposome.

## 4 Discussion

Current methods, such as vesicle transport assay, drug resistance/accumulation assay, and transwell assay, lack the capability to monitor real-time substrate transport and isolate the specific transport rate mediated solely by Pgp from the combined effects of multiple transporters present in the cell membrane. To address these limitations and adhere to FDA guidelines for investigating efflux transporters (Brum, 2020), this study introduces an *in vitro* transport assay using surface plasmon resonance (SPR). The SPR transport assay offers the advantage of assessing the Pgp substrate’s transport rate without requiring a subsequent measurement after the initial assay endpoint. Importantly, the SPR system employs purified Pgp reconstituted into liposomes, thereby excluding the influence of other transporters on efflux rate determinations. While conforming to FDA guidelines by utilizing the inside-out vesicle setup, the SPR assay effectively identifies Pgp substrates and quantifies their relative transport rates, aligning well with previous investigations conducted using established methods. Notably, this SPR transport approach, grounded in vesicle-reconstituted transporters, can extend its utility to the study of substrate efflux/influx mediated by other transporters, including BCRP9, OCT, OAT, and MATE, all mentioned in the FDA guidelines (Brum, 2020).

The relative transport rates of methadone and quinidine were assessed under conditions of ATP, ADP, and no nucleotide. The signal amplitudes differed significantly between the presence of 5 mM ADP and the absence of nucleotide. Additionally, the signal amplitude with ADP was notably lower than without nucleotide, suggesting distinct Pgp functionality with ADP compared to no nucleotide. This discrepancy is likely the result of ADP inducing a specific Pgp conformation that hinders substrate expulsion, as previous research proposed that an inward-facing Pgp-ADP conformation is less conducive to efficient efflux than the outward-facing conformation (Verhalen et al., 2017; Gibbs et al., 2018; Nguyen et al., 2020). In the absence of nucleotide, Pgp’s conformational flexibility allows substrate movement. This effect exhibits substrate-dependence, observed with methadone and quinidine, but not with desipramine, in our study.

In addition to assessing Pgp transport rate, the SPR technique also offers valuable insights into the real-time kinetics of Pgp transport. This not only enhances the comprehension of Pgp-mediated transport but also lends support to existing models elucidating the mechanism behind Pgp-mediated transport. The two models under consideration in this study are the vacuum cleaner and flippase models, both of which propose distinct pathways through which Pgp expels substrates. The vacuum cleaner model suggests that Pgp effluxes substrates from the inner leaflet of the lipid bilayer to the extracellular space, while the flippase model proposes expulsion from the inner leaflet to the outer leaflet of the bilayer (Sharom, 2014). By combining SPR data and COPASI modeling, our study utilizing mouse Pgp reconstituted in liposomes lends strong support to the vacuum cleaner model, bolstering it over the flippase model.

Both of these models find support in both biochemical and structural investigations. The vacuum cleaner model finds substantiation in the crystal structures of *h*Pgp in an outward-facing conformation and *m*Pgp in an inward-facing conformation (Li et al., 2014; Kim and Chen, 2018). The inward-facing conformation of *m*Pgp implies that the lateral opening is unoccupied when exposed to the inner leaflet. Conversely, the outward-facing conformation of *h*Pgp, observed post substrate translocation, demonstrates that the lateral opening remains situated within the inner leaflet. This consistent behavior, whereby the lateral opening of Pgp closes towards the outer leaflet throughout the substrate transport process, lends significant credence to the vacuum cleaner model as opposed to the flippase model.

Conversely, there exists evidence supporting the substrate flippase model. Investigations have indicated that Pgp can effectively transport a range of lipids, encompassing phosphatidylcholine, phosphatidylethanolamine, and sphingomyelin (van Helvoort et al., 1996; Bosch et al., 1997; Helvoort et al., 1997; Abulrob and Gumbleton, 1999; Pohl et al., 2002). The application of fluorescent labeling to these substrates has revealed a flip-flop transport mechanism from the inner leaflet to the outer leaflet (Romsicki and Sharom, 2001). Furthermore, an EPR study employing spin-labeled verapamil has lent support to the substrate flippase model. This study demonstrated that within the inside-out proteoliposome system, the spin-labeled verapamil was initially captured by Pgp in the outer leaflet and subsequently transported to the inner leaflet, exhibiting an apparent turnover rate of 5.8/s (Omote and Al-Shawi, 2002).

The kinetics data derived from the SPR transport assay for the three compounds, namely, desipramine, methadone, and quinidine, provided intriguing insights into the behavior of the Pgp transport system. Notably, all three compounds exhibited a clear preference for binding to the outer leaflet of the membrane compared to interacting with Pgp or diffusing into the inner leaflet. This observation suggests that the outer leaflet could play a significant role in driving the passive diffusion of chemicals into cells. However, once binding with Pgp occurred, the substrates were actively expelled into the core of the proteoliposome, indicating that Pgp possessed the ability to overcome the passive diffusion of these drugs.

The SPR transport method developed in this study is constrained by the localized SPR technology employed. This approach hinges on alterations in sensor absorbance when interactions transpire on the sensor surface. Consequently, it is unable to detect molecules with absorbance akin to that of the sensor, positioned around the 500 nm wavelength. Nonetheless, conventional SPR, reliant on changes in reflectance angle rather than absorbance, can identify these molecules. Furthermore, the study’s employment of mouse Pgp reconstituted in liposomes, as opposed to human Pgp, presents another limitation. Consequently, the delineated kinetics are applicable solely to mouse Pgp. However, given the substantial 80% sequence similarity between mouse and human Pgp, insights gleaned from investigations involving mouse Pgp still offer valuable understanding into the functioning of human Pgp.

Undoubtedly, this study has pioneered a novel approach for quantifying both Pgp efflux rate and Pgp efflux kinetics through the utilization of SPR. The primary objective was to align with the FDA guidelines while simultaneously addressing the limitations inherent in prevailing *in vitro* transport techniques. Most notably, this study stands as the pioneering effort to dissect the intricate kinetics of Pgp transport into distinct and detailed stages.

## Figures and Tables

**TABLE 1:** The relative transport rate of methadone, desipramine, and quinidine was measured using SPR and extracted from the literature. n=5. Error bar is ±SD.

	Literature	Experimental	Methods used to measure Pgp efflux ratio in literature
Methadone	1.9 (MDR-MDCK) 2.4 (Caco-2)	1.31 ± 0.1	Transwell system with Caco-2 or MDR1-MDCK cells Tournier et al. (2011)
Quinidine	10	1.64 ± 0.12	Transwell system with Caco-2 or MDR1-MDCK cells Patil et al. (2011)
Desipramine	2	1.30 ± 0.08	Drug accumulation assay with MDR1-MDCKII cells Doan et al. (2002)

**TABLE 2:** The kinetics from the SPR curve fitted to the vacuum cleaner model for three Pgp substrates: desipramine, methadone, and quinidine. n=5.

	Desipramine		Methadone		Quinidine	
	Kon (M^−1^s^−1^)	Koff (s^−1^)	Kon (M^−1^s^−1^)	Koff (s^−1^)	Kon (M^−1^s^−1^)	Koff (s^−1^)
Diffuse from outside to outer leaflet	1.15 × 10^−6^	4.1 × 10^−6^	5.07 × 10^−5^	8.34 × 10^−6^	4.62 × 10^−8^	1.20 × 10^−5^
Binding to Pgp	8.9 × 10^−3^	129	1.61 × 10^−6^	6.39 × 10^5^	6.69 × 10^−4^	823
Pgp transport to inside the proteoliposome	3.57 × 10^−5^	6.44 × 10^−8^	2.75 × 10^−4^	1.89 × 10^−4^	1.6 × 10^−3^	7.91 × 10^−8^
Diffuse from outer leaflet to inner leaflet	4.00 × 10^−5^	2.68	1.83 × 10^−7^	8.81 × 10^−7^	0.01	164
Diffuse from inner leaflet to inside the proteoliposome	1.10 × 10^−5^	1.09	0	493	1.1 × 10^−3^	0.13

## Data Availability

The raw data supporting the conclusion of this article will be made available by the authors, without undue reservation.

## Abbreviations:

AMPPNP: adenosine 5’-(β,γ-imido)triophsophate
CHAPS: 3-((3-cholamidopropyl) dimethylammonio)−1−propanesulfonate
COPASI: complex pathway simulator
DDM: *n*-dodecyl-β-*D*-maltoside
DEAE: diethylaminoethyl cellulose
FDA: Food and Drug Administration
KPi: potassium phosphate
NATA: N-acetyl-L-tryptophanamide
NBD: nucleotide-binding domain
Ni-NTA: nickel-nitrilotriacetic acid
Pgp: P-glycoprotein
*Pi*: inorganic phosphate
SPR: surface plasmon resonance
